# Pharmaceutical Excipients and Drug Metabolism: A Mini-Review

**DOI:** 10.3390/ijms21218224

**Published:** 2020-11-03

**Authors:** Rahul Patel, James Barker, Amr ElShaer

**Affiliations:** Drug Discovery, Delivery and Patient Care (DDDPC), School of Life Sciences, Pharmacy and Chemistry, Kingston University, Kingston upon Thames, Surrey KT1 2EE, UK; k1657774@kingston.ac.uk (R.P.); J.Barker@kingston.ac.uk (J.B.)

**Keywords:** Pharmaceutical excipients, metabolism, cytochrome P450

## Abstract

Conclusions from previously reported articles have revealed that many commonly used pharmaceutical excipients, known to be pharmacologically inert, show effects on drug transporters and/or metabolic enzymes. Thus, the pharmacokinetics (absorption, distribution, metabolism and elimination) of active pharmaceutical ingredients are possibly altered because of their transport and metabolism modulation from the incorporated excipients. The aim of this review is to present studies on the interaction of various commonly-used excipients on pre-systemic metabolism by CYP450 enzymes. Excipients such as surfactants, polymers, fatty acids and solvents are discussed. Based on all the reported outcomes, the most potent inhibitors were found to be surfactants and the least effective were organic solvents. However, there are many factors that can influence the inhibition of CYP450, for instance type of excipient, concentration of excipient, type of CYP450 isoenzyme, incubation condition, etc. Such evidence will be very useful in dosage form design, so that the right formulation can be designed to maximize drug bioavailability, especially for poorly bioavailable drugs.

## 1. Introduction

**Figure 1:**
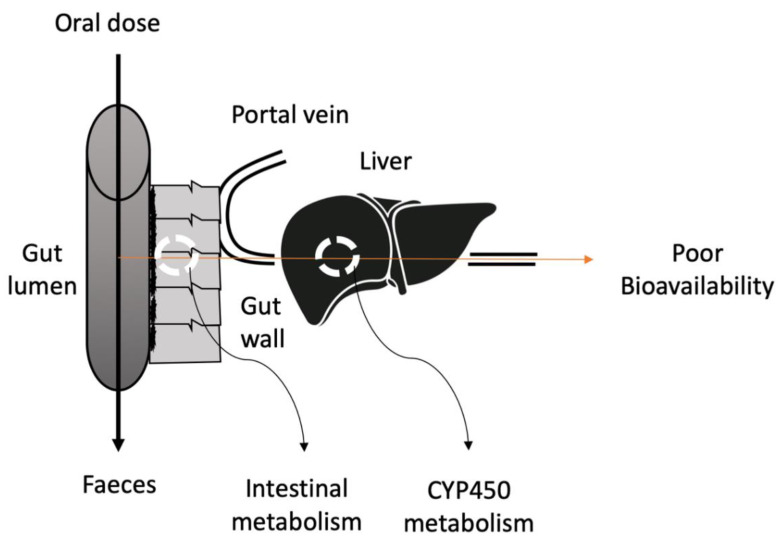
Schematic diagram depicting the route of poor bioavailability after oral administration of the drugs.

The most popular route for drug delivery is oral administration because of pain avoidance, ease of ingestion, patient compliance and versatility of drug candidates. Moreover, the manufacturing for oral drug delivery systems is less expensive as the production process is simple and there are no requirements for sterile conditions [1]. The growth rate of the oral drug delivery market between 2010 and 2017 was 10.3% [2]. Despite all the benefits of oral delivery, poor bioavailability of oral formulations is a limiting factor that can alter the efficacy and therapeutic effect [3]. Various factors are contributing to low oral bioavailability including physiological factor, high gastric emptying time, the effect of food, intestinal barrier and enzymatic degradation of drugs (Table 1). First-pass metabolism is one of the key factors responsible for poor bioavailability. The extensive metabolism of drugs prior to reaching the systemic circulation is known as the first-pass metabolism. After oral administration, the drug is absorbed by the gastrointestinal tract (GIT) and transported to the liver through the portal veins. Then, the drug is metabolized in the liver before reaching systemic circulation, resulting in a low available concentration at the intended target site (Figure 1). Due to insufficient plasma concentrations, the bioavailability of the drug is significantly reduced and therefore a high dose of the drug is required [4].

The principle enzymes responsible for first-pass metabolism or biotransformation of drug molecules are cytochrome P450 (CYP450). CYP450 includes a superfamily of hemeproteins which is categorized into families and subfamilies based on amino acid sequence homology [31]. They are allotted a family number (for instance, CYP1 or CYP2) followed by a subfamily letter (e.g., *CYP1A* or *CYP2C*) and are distinguished by a number for the individual enzyme or isoforms (e.g., *CYP1A2* or *CYP2C19*) [31]. According to Coller et al., the most commonly prescribed drugs in the United States (USA) are reported to be metabolized by CYP1, CYP2 and CYP3 families. The most common enzymes responsible for oxidation of approximately 79% of these drugs are CYP2C9, CYP2D6, CYP2C19 and *CYP3A4/5* [32].

## 2. Traditional Strategies to Overcome Pre-Systemic Metabolism

Several novel, as well as traditional, methods are being investigated to circumvent drug metabolism. Prodrugs, enzyme inhibitors, polymeric excipients, self-emulsifying drug delivery systems (SEDDS) and liposomes have been investigated over the years as inhibitors to metabolic enzymes.

### 2.1. Prodrug Approaches

Prodrug is one of the common approaches investigated, according to the literature. Approximately 10% of the drugs in the pharmaceutical market are categorized as prodrugs and one third of small molecular weight (Mw) drugs are classified as prodrugs [33,34,35,36,37,38]. Prodrugs are pharmacologically inert substances which undergo conversion, once inside the body, to release the active ingredient for its therapeutic effect [39,40]. Prodrugs successfully overcome the drug physicochemical and biopharmaceutical obstacles, thus enhancing their pharmacokinetic properties such as oral absorption and metabolism [41,42].

### 2.2. Enzyme Inhibitors

Another promising strategy to reduce the pre-systemic metabolism is the co-administration of CYP450 inhibitors with orally administered drugs [43]. Probably, the best-known enzyme inhibitor is grapefruit juice, which significantly improves the oral bioavailability of many drugs [44,45]. Food–drug interaction is a common occurrence and can be significant when the drug’s pharmacokinetics are altered. The classic example is the interaction between grapefruit juice and felodipine. Felodipine is known to undergo high pre-systemic metabolism resulting in very low absolute bioavailability with an average of 15%. A study showed the inhibitory effect of grapefruit juice flavonoids such as quercetin, naringenin and naringin on *CYP3A4*, using felodipine as a substrate [45]. The concentration-dependent inhibition was examined when the flavonoids were co-incubated with felodipine [45]. Moreover, many studies reported furanocoumarins, present in grapefruit juice, as an inhibitor for CYP450 [46,47,48,49,50,51,52,53,54]. Bergamottin inhibits *CYP1A2, CYP1B1, CYP 2A6, CYP2B6, CYP2C9, CYP2C19, CYP2D6, CYP2E1, CYP3A4* and *CYP3A5*. The dihydroxybergamottin (DHB) and paradisins inhibit *CYP1A2, CYP1B1, CYP2C9, CYP2C19, 2D6* and *CYP3A4* [55].

Besides grapefruit juice, many drugs in the market such as ketoconazole, Troleandomycin, saquinavir, diltiazem and fluconazole exhibit an enzyme inhibitory effect resulting in improved bioavailability of many drugs [43,56]. For instance, the oral bioavailability of alfentanil was increased 19-fold with troleandomycin [57]. However, co-administration of enzyme inhibitors can lead to adverse drug effects which also includes fatal events. For example, when clarithromycin (inhibitor) is co-administered with astemizole, terfenadine or cisapride (substrates), a severe ventricular arrhythmia may occur [58].

## 3. Other Approaches

These include polymeric excipients, self-emulsifying drug delivery systems (SEDDS) and liposomes. Normally, polymers are used as enteric coatings for different types of drugs to overcome hydrolytic instability [59]. Some polymers, such as polycarbophil and carbomer, demonstrate inhibitory effects on trypsin, which decrease pre-systemic metabolism in the intestine. That means drugs incorporated in such polymers can be protected from pre-systemic degradation by trypsin [60]. Similarly, most of the studies on SEDDS and liposomes focus on increased solubility by these systems and their protective property towards pre-systemic metabolism is hardly examined [43].

### 3.1. Novel Approach to Overcome Pre-Systemic Metabolism

The addition of commonly-used pharmaceutical excipients can be a potential solution to these problems. Pharmaceutical excipients are ingredients other than the active pharmaceutical ingredient (API) present in a finished pharmaceutical drug formulation. These are frequently used as lubricants, diluent, binders, flavorings, coating and coloring agents for the formulation. These substances are often therapeutically inert [61].

The functional roles of pharmaceutical excipients include modulating bioavailability and solubility of APIs, increasing the stability of APIs in the dosage form, maintaining the osmolarity and/or pH of the liquid formulations, preventing dissociation and aggregation, etc. Recently, addition of pharmaceutical excipients in drug formulations have gained attention which can alter the pharmacokinetics of drugs, resulting in improved bioavailability [61]. The aim of this review article is to survey the current literature and evaluate the effect of different pharmaceutical excipients on metabolic enzymes. This review looks at the effect on surfactant, polymers, etc. excipients on the expression of cytochrome P450 enzymes.

### 3.2. Effect of Excipients

Substances that can enhance or inhibit cytochrome P450 activity can alter the rate of drug metabolism, leading to an increase or decrease in drug bioavailability. There are many studies on new materials as drug delivery vehicles, including vesicles, block copolymer micelles, degradable polymer particles, dendrimers, polymer prodrugs and lipid nanoparticles; however, the effect of excipients used is often ignored. For instance, liquid acetaminophen, which contains propylene glycol, is less toxic than solid preparations with no propylene glycol. The main reason for acute hepatic failure in Europe and the United States is acetaminophen [62,63]. Its toxicity is due to the reductive metabolism via *CYP2E1* [64]. Liquid formulation of acetaminophen contains a solubilizing agent called propylene glycol, which is used to dissolve the drug in aqueous solution [65]. Since children ingest liquid formulation and are less vulnerable to its toxicity, a single-blinded cross-over study was conducted to compare metabolism of solid and liquid acetaminophen 15 mg/kg dose by *CYP2E1*, using 15 healthy adults as volunteers. As a result, the measured AUCs for the metabolites were 16% lower than solid formulation. This is because propylene glycol is a competitive antagonist to *CYP2E1*; hence, it shows the protective effect in liquid formulation [65]. 

One method by which excipients may alter the drug metabolism is by inhibiting CYP450 enzymes present in cellular microsomes [61].

### 3.3. Surfactants 

Surfactants, also known as surface-active agents, possess hydrophilic (polar) and hydrophobic (non-polar) characteristics. The hydrophobic (non-polar) part is referred to as the tail group and the hydrophilic (polar) part as the head group. Surfactants are normally used to increase the solubility of the drugs and to decrease the interfacial tension between the drug and the medium [66]. Surfactants can be categorized into four different groups: anionic, cationic, zwitterionic and non-ionic surfactants (Table 2). Anionic surfactants carry a negative charge, whereas cationic surfactants carry a positive charge on their hydrophilic head. Zwitterionic surfactants have the potential to carry both positive and negative charges [66]. 

Cremophor EL is a heterogeneous non-ionic surfactant made up of castor oil and ethylene oxide with a molar ratio of 1:35 [67]. Cremophor EL helps in solubilizing many hydrophobic drugs which include photosensitizers, immunosuppressive agents, sedatives, anesthetics and anticancer drugs (experimental) [67]. Different in vitro studies have been reported for the impact of Cremophor EL on the metabolism of drugs [68,69,70]. In 2010, a study focused on the effect of Cremophor EL on *CYP3A4* and *CYP2C9* mediated metabolism of testosterone and diclofenac, respectively [68]. Cremophor EL was tested at different concentrations (0.01–100 mM) using human liver microsomes in vitro. As a result, the inhibitory effect of Cremophor EL was found to be concentration dependent. The half-maximal inhibitory concentration (IC_50_) of the *CYP3A4*- and *CYP2C9*-mediated metabolism were determined as 0.60 and 0.03 mM, respectively. 

Mudra et al. further showed that solubilizing agents inhibited verapamil-N-demethylase activity in vitro and in situ. The rate of verapamil-N-demethylation was reduced in the presence of Cremophor EL, suggesting moderate inhibition of *CYP3A4*: 20.7% and 21.8%, in situ and in vitro at 47.5 μg/mL, respectively [69]. The inhibition of CYP450 by polyethoxylated solubilizing agents (e.g., Cremophor EL, Tween 80) can be attributed to the collective evidence that supports the following hypothesis: The drug absorption is altered in the presence of polyethoxylated solubilizing agents due to agent-produced membrane fluidization, causing in local environment perturbation required for protein to function [70,71,72,73]. Similarly, the outcomes in this article are reliable with agent-induced fluidization of microsomal membrane resulting in perturbation of the enzyme micro-environment, thus decreasing *CYP3A4* function.

To support this hypothesis, another study on Cremophor EL presented results consistent with the previous studies. In vitro metabolism of 7-ethoxycoumarin was studied at three different concentration of the surfactant (0.03%, 0.06% and 0.1% *w/v*). Various enzymes are responsible for metabolism of ethoxycoumarin, but the major enzymes are *CYP450 1A2*, *CYP450 1A1* and *CYP450 2B*. Increasing the concentration of Cremophor EL decreased the metabolic activity of these enzymes [68,74]. The CYP450 activities were reduced from 97% to 93% when surfactant concentration was increased from 0.03% to 0.10%. This is because the surfactant concentration above its critical micellar concentration (CMC) value disrupts the CYP450 enzymes membrane, causing an inhibitory effect. In contrast, below its CMC value, there is less disruption [74].

In 2004, Gonzalez et al. investigated the effect surfactants have on the metabolism of the *CYP3A2* substrate by Midazolam (MDZ) by determining the intrinsic clearance (Clint) of MDZ in rat hepatocyte and rat microsome systems [75]. In the rat hepatocytic system, the Clint of MDZ decreased significantly above its critical micellar concentration (CMC) at 0.03% (Clint/0.03% = 70.3% ± 2.1%) and 0.3% (Clint/0.3% = 54.9% ± 2.2%), whereas the Clint of MDZ was not altered significantly below its CMC (at 0.0003% and 0.003%). Similar outcomes were observed with rat liver microsomes; significant decrease in Clint of MDZ at 0.03% (Clint/0.03% = 89.5 ±3.7 µL/min/mg protein) and 0.3% (Clint/0.3% = 35.0 ±0.8 µL/min/mg protein) of the Cremophor EL. Thus, the addition of Cremophor EL reduced the intrinsic clearance of MDZ by inhibiting CYP450 in both systems [75].

Taxol is an active ingredient used for refractory ovarian cancer, lung cancer and breast cancer. It is readily metabolized by the CYP450 system in the liver. A study conducted by Carlos et al. (1994) illustrated that Cremophor EL had the most significant effect on taxol metabolism, by the CYP450 system, compared to other co-administered drugs (diphenhydramine, cimetidine and dexamethasone). In human and rat liver microsomes, the formation of 6α-hydroxytaxol was completely prevented by Cremophor EL at 20 µL/mL. In human liver slices, Cremophor EL reduced the formation of 6α-hydroxytaxol as well as the ratio of metabolite to parent drug at 20 µL/mL [76]. However, at 2 µL/mL, Cremophor EL showed very little effect. These results suggest that Cremophor EL indirectly reduces the taxol uptake by the liver. To conclude from all the studies, the inhibition of CYP450 enzymes by Cremophor EL seems to be dependent on the concentration of surfactant, type of isoenzyme and type of microsomal assay.

Cremophor RH-40 is found in many oral drug formulations, which is mainly used to improve solubilization of the drugs, such as Tegretol, Anafranil and Sandimmun Neoral. Cremophor RH-40 has a different molar content of ethylene oxide (45 mol) than Cremophor EL [77]. The inhibition of CYP450 enzymes by Cremophor RH-40 has been reported in many studies [63]. Christiansen et al. examined the effect of non-ionic surfactants on *CYP3A4* and *CYP2C9* enzymes [68]. The inhibitory action of Cremophor RH40 (IC50 = 0.80 mM) was found to be less than Cremophor EL (IC_50_ = 0.60 mM) for *CYP3A4*, whereas stronger inhibition was reported with a similar IC_50_ value (0.03 mM) for *CYP2C9*. To understand the action of Cremophor RH40 in biotransformation, it was examined for its inhibitory effect on CYP3A4 in vitro as well as in vivo [61]. According to the in vitro study conducted by Ren et al. (2008), the inhibition rate of *CYP3A4* by Cremophor RH40 was 99.40%. Furthermore, single and multiple doses of Cremophor RH40 were examined in male Sprague-Dawley rats for its impact on MDZ metabolism. In comparison to control saline, Cremophor RH40 increased the area under curve (AUC_0-∞_) of MDZ up to 1.1-fold and decreased the production of 1′-Hydroxymidazolam (1′-OH-MDZ) up to 0.44-fold in a single dose. The MDZ AUC_0-∞_ was increased by 1.69-fold and 1’-OH-MDZ AUC_0-∞_ was decreased by 0.9-fold in a multiple-dose regimen [61].

These results are consistent with the recent study on a Cremophor RH40-based self-micro emulsifying drug delivery system (SMEDDS) [78]. A significant decrease in 1′-OH-MDZ production was observed (*p* < 0.05), when the concentration of Cremophor RH40 was increased from 0.05% to 3% w/v. Studies have revealed that chemotherapeutic substances can downregulate the *CYP3A* gene expression [79,80]. Therefore, Western blot analysis was performed to examine the effect of SMEDDS on the *CYP3A* activity. As a result, Cremophor RH40-based SMEDDS reduced the *CYP3A* protein expression levels at dilutions ranging from 1:50 to 1:100. Another study reported a similar significant inhibition of *CYP3A4* and *CYP2C19* enzymes in vitro by Cremophor RH40 when rabeprazole was used as a probe drug (*p* < 0.05) [81]. It was concluded that these inhibitory effects involved direct action or micelle formation to disrupt CYP activities.

Recently, Tween 80 has been reported to inhibit different isoenzymes of CYP450: 3A4, 2C9, 1A1, 1A2 and 2B. Tween 80 (polysorbate 80) is the most commonly-used hydrophilic non-ionic surfactant with the ability to improve the solubility of compounds [69]. Tween 80 is derived from oleic acid and polyethoxylated sorbitol and has been used in preclinical and clinical drug formulations [69]. Moderate inhibition of *CYP3A4* and *CYP2C9* by Tween 80 was determined. The IC_50_ value for *CYP3A4* and *CYP2C9* was found to be 0.40 and 0.04 mM, respectively. Christiansen et al. and Rao et al. illustrated the inhibition of CYP450 to be a concentration-dependent manner. For example, when the concentration of Tween 80 in SMEDDS was increased from 0.05% to 3% (*w/v*), the 1′-OH-MDZ production was reduced from ~80% to 30%. The in vitro metabolism of 7-ethoxycoumarin was reduced from 85% to 65% when the concentration of Tween 80 was increased from 0.03% to 0.10% [68].

The incubation of Tween 80 with rat hepatocytes reduced Clint of MDZ significantly, above its CMC (Clint/0.03% = 75.2% ±1.6%; Clint/0.3% 79.2% ±1.5%, *p* < 0.05), whereas the inhibitory effect of Tween 80 was reported below its CMC with rat liver microsomes (Clint/0.003% = 70% ± 5.7%, Clint/0.03% = 66.9% ± 1.0%, Clint/0.3% = 8.24% ± 0.28%, *p* < 0.05) [78]. A similar study reported the inhibition of *CYP3A4* by approximately 20% with a Tween 80 concentration of 50 mM [61]. Mudra et al. measured the impact of Tween 80 on intestinal verapamil-N-demethylation activity in-situ and in vitro. The inhibition rate was found to be 56.3% (in-situ) and 13.5% (in vitro) at 25 μg/mL, reflecting the reduction of *CYP3A* activity [69].

Tween 20 (Polysorbate 20) and Tween 80 are composed of the same hydrophilic group but different hydrophobic groups. Tween 20 has a mixture of palmitic, stearic, lauric and myristic acids, whereas Tween 80 contains oleic, linoleic and stearic acids [61]. According to Ren et al. and Randall et al., Tween 20 is a more potent inhibitor than Tween 80. The production of 1′-OH-MDZ was inhibited by around 80% at 50 mM Tween 20 concentration. It also decreased the metabolism of 7-ethoxycoumarin from 66% to 56% at a concentration ranging from 0.03% to 0.10% *v/v* [61,74].

Triton X-100 (TX-100) is an octylphenol polyethoxylated non-ionic surfactant. Its structure consist of poly(ethylene glycol) with a 4-(1,1,3,3-tetramethylbutyl)phenyl group [82]. TX-100 has been reported as a strong inhibitor for CYP450 enzymes. It reduced the metabolism of CYP3A4 substrates: 7-ethoxycoumarin by 54% and midazolam by 99.8% [74,81]. The interaction of TX-100 with CYP450 in Prochilodus scrofa was studied using antioxidant and mono-oxygenase system. The CYP content at 0.05 mM of TX-100 was found to be 0%, explaining the stronger inhibition of CYP450. Similarly, competitive inhibition of *CYP1A1* by TX-100 was reported far below its CMC value (250 μM) [83].

Furthermore, multiple studies pointed out the ability of other surfactants to interfere with CYP450, which can lead to improved bioavailability of drugs. The surfactants that were reported to show inhibitory actions are listed in Table 2. 

### 3.4. Polymers

Polymers are macromolecular compounds and constitute a large and diverse group of substances, including synthetic polymers, semi-synthetic polymers, natural polymers and fermentation products (Table 3). These polymers are commonly used as excipients in pharmaceutical dosage forms; parenterally, orally, nasally, rectally, intravaginally, inhalationally and topically, on the oral mucosa and in ophthalmic preparations [84].

Lihui Qiu et al. evaluated the in vitro inhibition of six CYP isoforms by different mPEGx-PCLx (methoxy poly(ethylene glycol)-poly(ε-caprolactone)) amphiphilic copolymer micelles: mPEG2k-PCL2k, mPEG2k-PCL3.5k, mPEG2k-PCL5k and mPEG2k-PCL10k. The inhibitory effect was found to be concentration-dependent in manner [85]. All CYP450 enzymes were significantly inhibited by mPEG2k-PCLx when the concentration was increased to 1000 μg/mL. For example, the CYP activities were reduced to 68.7% for *CYP2D2*, 64.6% for *CYP2C6*, 40.4% and 33.5% for *CYP3A2/1*, 38.1% for 2B1, 26.2% for 2C11 and 9.4% for 1A2 by mPEG2k-PCL2k at 1000 μg/mL. The extent of inhibition, ranked downwards, was as follows: mPEG2k-PCL2k > mPEG2k-PCL3.5k > mPEG2k-PCL5k > and mPEG2k-PCL10k [85]. 

Similarly, Martin and co-workers investigated the effect of 10 commonly-used polymers on seven CYP isoforms (2E1, 3A4, 3A5, 2C9, 2C19, 1A2 and 2D6). As shown in Table 4, nine out of ten polymers inhibited CYP activities. Cytochromes 2E1, 3A5, 2C9, 2C19 and 2D6 were inhibited by polyethylene glycol (PEG), while 2E1, 3A4, 3A5 and 2C9 were downregulated by pluronic F68. Pluronic F127, polyvinyl acetate (PVA) and sodium carboxymethyl cellulose (NaCMC) inhibited 2E1, 2E1 and 1A2, respectively. Hydroxypropyl methylcellulose (HPMC) inhibited 2E1 and 3A5, whereas polyvinyl pyrrolidone (PVP) downregulated 3A4 and 1A2. Other polymers, such as Kollicoat, in this study also inhibited specific enzymes, apart from hydroxypropyl cellulose (HPC), which exhibited no effect on any of the enzymes [86].

Huang et al. used the Hill equation and Lineweaver–Burk plots to determine the inhibitory effect of pluronic F68 (F68) on *CYP3A4*. The dose-dependent inhibition was reported with the Ki and IC_50_ values for F68 averaged 0.16 and 0.11 mg/mL, respectively [87]. The mechanism of action was based on the previously reported inhibitory effect study, which includes the direct interaction with CYP450 enzymes, cell membrane disruption and alteration of cell membrane [75].

Another study tested 22 commonly used excipients, which included sodium alginate, PEG4000, PEG1000, PEG6000 and PEG 2000. All the listed polymers inhibited 1′-OH-MDZ formation in the following order (from strong to weak): Sodium alginate > PEG4000 > PEG1000 > PEG6000 > PEG2000 [61]. The physical and chemical nature of each excipient clearly play a major role in their inhibitory capacity. Thus, sodium alginate was the most effective inhibitor compared to the other polymers due to its ability to disrupt *CYP3A4* activity strongly. On the other hand, the outcomes of this study were limited to *CYP3A4*. As previously mentioned, there are many families and subfamilies of CYP450. There is a possibility that the least effective polymer (PEG2000) could be the most effective on other CYP450 isoenzymes.

Pluronic P85 (P85) is a block copolymer consisting of two equal polyoxyethylene chains joined by a polyoxypropylene chain. A study revealed that P85 strongly inhibited norverapamil formation by *CYP3A* in a concentration-dependent manner [88]. The study included enterocyte-based metabolism, where the P85 concentrations used were 0.01% and 0.1% *v/v*. Compared to other polymers in the study, P85 reduced the formation of norverapamil by around 25% and 50% at 0.01% and 0.1% *v/v*, respectively [88].

### 3.5. Fatty Acids

Fatty acids (FA) are the main components of phospholipids, triacylglycerols and many complex lipids. FAs are present in the majority of dietary fat in humans. Different foods provide a variety of fatty acids. In addition, the human body can synthesize them, either from other fatty acids or nonlipid precursors, e.g., glucose. In recent years, studies have revealed that various FAs have a wide range of microbicidal activity against several Gram-positive and Gram-negative bacteria, as well as enveloped viruses, including *S. aureus* and *N. gonorrhoeae* [89,90]. For instance, lauric acid (LA) was found to be the most potent inhibitor for the growth of Gram-positive bacteria. These direct or indirect inhibitory effects are due to the destabilization of the bacterial cell membrane caused by fatty acids [91,92,93,94,95]. 

Besides their potent microbicidal activity, FAs can also reduce pre-systemic metabolism of drugs by inhibiting CYP450 enzymes. The effect of saturated and unsaturated fatty acids on CYP3A4, 2E1, 2D6, 2C19, 2C9, 2C8, 2B6, 2A6 and 1A2 were studied using midazolam 1-hydroxylation, chlorzoxazone 6-hydroxylation, dextromethorphan O-demethylation, mephenytoin 4-hydroxylation, diclofenac 4-hydroxylation, amodiaquine N-deethylation, bupropion hydroxylation, coumarin 7-hydroxylation and phenacetin O-deethylation, respectively. As a result, out of all the saturated fatty acids, lauric acid showed potential inhibition of 2B6. Myristic acid inhibited 1A2 (IC_50_ = 15.8 μM), 2B6 (IC_50_ = 10.7 μM), 2C8 (IC_50_ = 13.3 μM) and 2C9 (IC_50_ = 36.1 μM). One of the mono-unsaturated fatty acids, oleic acid, inhibited all the CYP isoforms except for *CYP2E1*, *CYP2C19* and *CYP2A6*. Similarly, arachidonic acid inhibited activities of *CYP3A4*, *CYP2C19, CYP2C9, CYP2C8, CYP2B6* and *CYP1A2*. Other FAs also showed a distinct inhibitory effect on different isoforms: gondoic acid inhibited all except 2C8; linoleic acid inhibited *CYP2B6*, *CYP2C8* and *CYP2C9*; linolenic acid inhibited *CYP1A2*, *CYP2B6*, *CYP2C8* and 2C9; and timnodonic acid inhibited 2C8 (Table 5) [96]. Amongst all the isoenzymes, the majority of FAs inhibited *CYP2C8* activity. This can be attributed to the large active site on *CYP2C8*, which allows different sized substrates to accommodate. It also has a peripheral FA binding site that can alter the dynamics of the main active site, affecting the reaction catalyzed by this enzyme. Moreover, it is responsible for the transformation of polyunsaturated FAs to epoxide products (signaling agents). Hence, the unsaturated fatty acids are potent inhibitors for CYP enzymes than saturated fatty acids [97,98].

The results are consistent with a published study on inhibitory effect of saturated and polyunsaturated fatty acids by Yao and co-workers. Two saturated (palmitic acid and stearic acid) and five polyunsaturated fatty acids (linoleic acid, linolenic acid, arachidonic acid, eicosapentaenoic acid and docosahexaenoic acid) were examined for their effect on six isoforms of CYPs (1A2, 3A4, 2C9, 2C19, 2E1 and 2D6). As shown in Table 6, saturated fatty acids showed no inhibitory effect, while all polyunsaturated fatty acids inhibited all six isoforms [99]. These polyunsaturated fatty acids had little inhibitory effect at low concentrations, whereas there was a complete inhibition of all isoforms at 200 μM. One potential explanation based on the results is that, at high concentration, polyunsaturated fatty acids disrupt the microsomal membrane, which prevents the binding of the drug to the active site of the CYP450 enzyme [99]. However, other studies have reported that the CYP enzymes can also catalyze the metabolism of polyunsaturated fatty acids. Thus, fatty acids can act as a common substrate for the active site and compete with drugs to bind with CYP enzymes [100,101]. Therefore, the mechanism of inhibition remains unknown.

## 4. Co-Solvents/Solvents

**Figure 2:**
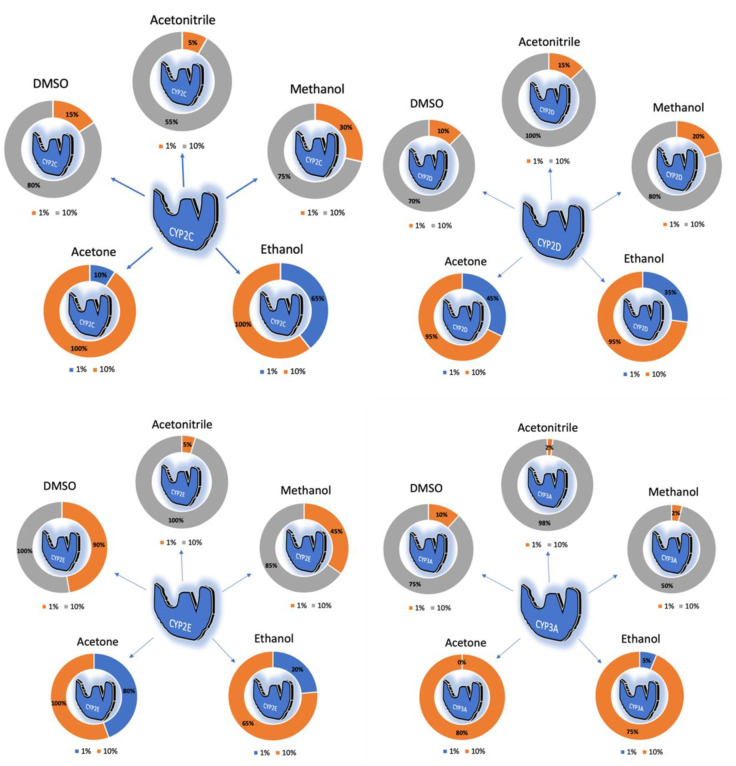
Illustrates the inhibition of *CYP2C, CYP2D, CYP2E, CYP3A and CYP1A* by acetonitrile, methanol, ethanol, acetone and DMSO at 1% and 10% concentrations.

In the last few decades, the effect of organic solvents (such as acetonitrile) on CYP-mediated metabolism has been reported by many researchers [102,103,104,105,106,107,108,109]. Most of the in vitro studies include organic solvents to dissolve the compounds and prepare the samples for analysis [110,111,112,113,114]. However, at higher concentrations, these organic solvents inhibit different isoforms of CYPs. Li et al. evaluated the inhibitory effect of five organic solvents (dimethyl sulfoxide (DMSO), acetonitrile, methanol, ethanol and acetone) on five CYP isoforms [115]. All organic solvents at 10% v/v inhibited CYPs activities, whereas >10% inhibition of *CYP2D* and >40% inhibition of *CYP2E* (except acetonitrile) was reported at 1% *v/v*. Overall, the organic solvents showed concentration-dependent inhibition. The inhibitory effect of organic solvents is summarized in Figure 2. DMSO and ethanol showed highest inhibitory effect amongst all organic solvents. 

Another study demonstrated similar results with DMSO. When diazepam was dissolved in DMSO, the *rCYP3A4* activity was inhibited in a mixed-type or competitive manner [116]. The inhibition of *rCYP3A4* was concluded to be concentration- and time-dependent. The DMSO inhibitory constant (Ki) value for the formation of temazepam and nordiazepam was found to be 24 and 6 mM, respectively.

In 2009, the effect of co-solvent on diclofenac and S-warfarin metabolism by *CYP2C9* was studied using ethanol [117]. The inhibitory effect was determined to be substrate dependent. The inhibition of S-warfarin metabolism required less ethanol concentration compared to diclofenac metabolism. Ethanol could inhibit S-warfarin metabolism at a low concentration of 17 mM (0.1% *v/v*), whereas it required 510 mM (3.0% *v/v*) of ethanol to competitively inhibit *CYP2C9*. Ethanol also presented concentration-dependent inhibition for both substrates: S-warfarin and diclofenac. 

**Figure 3:**
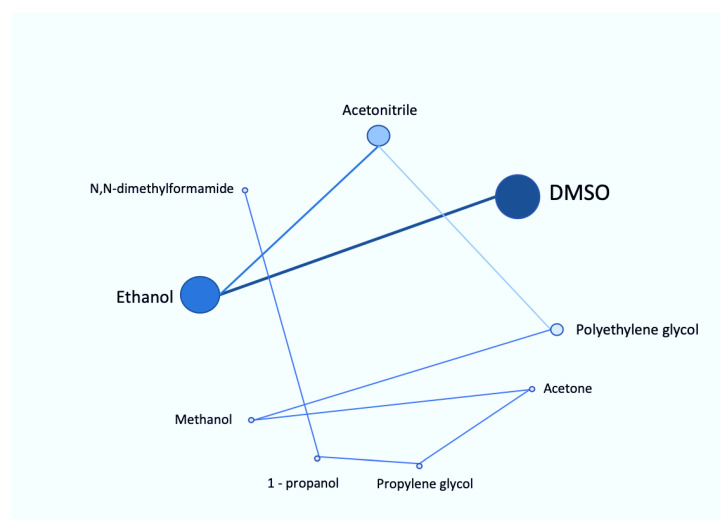
Roadmap of various reported organic solvents based on their inhibitory effect on CYP450 system. The size of each circle represents the potency of various organic solvents and the lines depict the order of inhibition: from DMSO being the most potent to N,N-dimethylformamide being the least.

Eight organic solvents have been studied for their effects on *CYP3A4* activity using pooled human microsomes [102]. The hydrophilic organic solvents used for this study were DMSO, acetonitrile, methanol, 1-propanol, ethanol, polyethylene glycol, propylene glycol and N,N-dimethylformamide. All organic solvents, except methanol, had significantly decreased testosterone 6β-hydroxylation activity, which reflected the inhibition of *CYP3A4*. In contrast, only DMSO and polyethylene glycol showed potential inhibitory effect on midazolam 1′-hydroxylation activity. Similar to this study, DMSO showed strongest inhibition for both metabolic activities. Moreover, the inhibition of testosterone 6β-hydroxylation activity by DMSO was in a concentration-dependent manner: from 10% to 50% inhibition at from 0.1% to 1% *v/v* concentration. The summary of all the reported organic solvents is shown in Figure 3.

## 5. Effect of Excipients on CYP450 Expression

As discussed above, the alteration of CYP450 activities by excipients were mainly through direct inhibition. However, studies also revealed that some excipients can alter the metabolic mechanism via mRNA/protein expression regulation [61,118], thus modulating absorption and metabolism of Class 3 drugs (high aqueous solubility and low intestinal permeability). Gene expression of the main metabolizing enzymes can be modulated by many environmental toxins or drugs, which can affect the toxicity and efficacy of co-medicated drugs and cause drug–drug interactions [119,120,121,122]. Recently, Tompkins et al. investigated the effect of nineteen excipients on regulation of *CYP3A4* expression in human liver and colon cells [118]. *CYP3A4* is a highly inducible isoenzyme and is mainly regulated by a xenobiotic receptor named Pregnane X receptor (PXR), at the transcription level [123,124]. This study also included a PXR activation assay to predict the effect of excipients on *CYP3A4* expression. As a result, all the excipients failed to activate the *CYP2B6* promoter used for PXR activation assay. Instead, few excipients (HPMC, pregelatinized starch and polysorbate-80) showed a decrease in the multi-drug resistance gene (MDR1) and *CYP3A4* expression (Table 7). These results suggest that excipients stimulate their effect via an alternate route.

Based on a previous study, Takeshita et al. evaluated the effect of plasticizers on PXR-mediated transcription by the luciferase receptor, PXR-coactivator interaction, PXR knockdown, *CYP3A4* activity assay and PCR analysis of *CYP3A4* enzyme expression [125]. Tompkins et al. (2010) used only two plasticizers which showed no effect on PXR or *CYP3A4* induction. However, this study was focused on eight pharmaceutical plasticizers: acetyl tributyl citrate (ATBC), acetyl triethyl citrate, tributyl citrate, triethyl citrate, diethyl phthalate, dibutyl phthalate, triacetin and dibutyl sebacate. As a result, ATBC, dibutyl phthalate and acetyl triethyl citrate activated PXR. ATBC being the most potent transcription inducer showed dose-dependent activation. However, ATBC induced only intestinal *CYP3A4* expression and not hepatic expression. This could be due to the PXR splice variants that vary in their gene targets, expression patterns, ligands, biological functions, subcellular localization and protein interactions [125]

## 6. Conclusions

Pharmaceutical excipients play an important role in pharmaceutical products and are often presumed to be pharmacologically inert. However, there is growing evidence that they can change the pharmacokinetics of APIs through various mechanisms, such as *P-gp* inhibition and CYP450 inhibition. In this review, we present recent research concerning the effects of common pharmaceutical excipients on pre-systemic metabolism by phase I metabolic enzymes (CYP450). According to our review, more than 40 commonly-used excipients were revealed to interfere with different isoforms of CYP450 in vitro, although very few have been assessed in humans. Based on the evidence, the mechanism of action was mainly found to be direct inhibition of the enzymes. Out of all the various excipients, surfactants were the most potent inhibitors due to their ability to cause perturbation of the enzyme’s microenvironment. Despite many similarities in the results from different articles, there appears to be a need for a robust approach to integrate the in vitro data that can predict pharmacokinetic changes in humans. Further research investigations are warranted to shed light on this issue.

## Figures and Tables

**Table 1:** Examples of drugs with very poor bioavailability with reported reasons.

Drugs	Pharmacological Class	Bioavailability (%)	Reasons	References
Alendronate	Bisphosphonates	0.59–0.78	Poor solubility and absorption	[5,6,7]
Atorvastatin	Statins	14	P-gp and CYP450 activities	[8,9,10]
Bromocriptine	Dopamine receptor agonists	5–10	Extensive first-pass effect	[11,12,13]
Clodronate	Bisphosphonates	1	Poor solubility and absorption	[5,6,7]
Cytarabine	Antimetabolites	20	Intestinal and hepatic first-pass	[14]
Domperidone	D2 receptor antagonists	15	Gut and liver first-pass	[15]
Doxorubicin	Anthracycline antibiotics	5	Hepatic and intestinal metabolism	[16]
Budesonide	Corticosteroids	11	Hepatic first-pass effect	[17]
Etidronate	Bisphosphonates	5	Poor solubility and absorption	[6,7,13]
Felodipine	Calcium channel blockers	15	P-gp and CYP450 activities	[17]
Isradipine	Calcium channel blockers	15	P-gp and CYP450 activities	[18]
Fluvastatin	Statins	20	P-gp and CYP450 activities	[8,9,10]
Nimodipine	Calcium Channel blockers	13	P-gp and CYP450 activities	[19]
Hyoscine	Antispasmodics	20	Hepatic metabolism	[20]
Ketamine	Dissociative anesthetics	20	Hepatic and intestinal metabolism	[21]
Lovastatin	Statins	<5	P-gp and CYP450 activities	[8,9,10]
Morphine	Opioids	20–33	Gut and liver first-pass	[22]
Pyridostigmine	Acetylcholinesterase inhibitors	14	Poor absorption	[23]
Naloxone	Opioid antagonists	2–10	Extensive first-pass but 90% absorption	[24]
Naltrexone	Opiate antagonists	5–40	First-pass, enterohepatic recycling	[10]
Pamidronate	Bisphosphonates	1	Poor solubility and absorption	[5,6,7]
Pravastatin	Statins	17–34	P-gp and CYP450 activities	[8,9,10]
Prochlorperazine	Phenothiazines	20	Intestinal and hepatic first-pass	[25]
Risedronate	Bisphosphonates	<1	Poor solubility and absorption	[5,6,7]
Selegiline	Monoamine oxidase type B inhibitors	20	Extensive first-pass	[26]
Simvastatin	Statins	5–48	P-gp and CYP450 activities	[8,9,10]
Sumatriptan	Serotonin receptor agonists	20	Hepatic first-pass	[27]
Tacrine	Cholinesterase inhibitors	10–30	Hepatic first-pass	[28]
Terbutaline	Adrenergic receptor agonists	9–21	Extensive first-pass and poor absorption	[29]
Lidocaine	Local anesthetics	3	Hepatic first-pass effect	[30]
Tiludronate	Bisphosphonates	6	Poor solubility and absorption	[5,6,7]

**Table 2:** Summary of the effect of surfactants on CYP activities.

Surfactants	Substrates	Mechanism of Action	Structures	Type	References
Brij 35	7-ethoxycoumarin	Increased *CYP3A4* inhibition with increased surfactant concentration	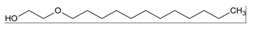	Non-ionic	[74]
Brij 58	Rabeprazole	Significant inhibition of drug degradation by CYP enzymes	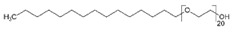	Non-ionic	[81]
CTAB	7-ethoxycoumarin	Increased *CYP3A4* inhibition with increased surfactant concentration	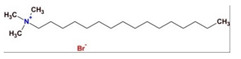	Cationic	[74]
Kollidon 12 PF	7-ethoxycoumarin	Increased *CYP3A4* inhibition with increased surfactant concentration	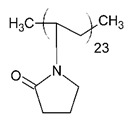	Non-ionic	[74]
Lutrol F68 NF	7-ethoxycoumarin	Increased *CYP3A4* inhibition with increased surfactant concentration	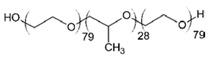	Non-ionic	[74]
Octyl-B-D-glucopyranoside	7-ethoxycoumarin	Increased *CYP3A4* inhibition with increased surfactant concentration	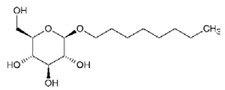	Non-ionic	[74]
SDS	7-ethoxycoumarin	Increased *CYP3A4* inhibition with increased surfactant concentration	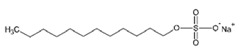	Anionic	[74]
Solutol HS 15	7-ethoxycoumarin	Increased *CYP3A4* inhibition with increased surfactant concentration	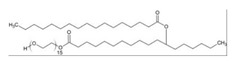	Non-ionic	[74]
Triton X-100 reduced	7-ethoxycoumarin	Increased *CYP3A4* inhibition with increased surfactant concentration	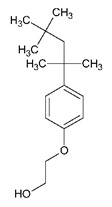	Non-ionic	[74]
Polysorbate 80	Testosterone Diclofenac	Increased *CYP3A4* and *CYP2C9* inhibition in concentration-dependent manner	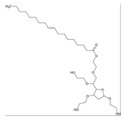	Non-ionic	[68]
TPGS	Testosterone Diclofenac	Increased *CYP3A4* and *CYP2C9* inhibition in concentration-dependent manner	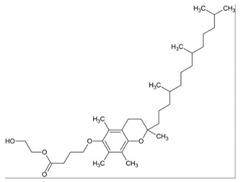	Non-ionic	[68]
Sucrose laurate	Testosterone Diclofenac	Increased *CYP3A4* and *CYP2C9* inhibition in concentration-dependent manner	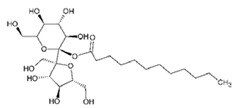	Non-ionic	[68]
Gelucire 44/14	Rabeprazole	Significant inhibition of drug degradation by CYP enzymes	Lauroyl polyoxyl-32 glycerides (C9H14N2)	Non-ionic	[81]
Polyoxyl 40 Stearate	Midazolam	Strong inhibition of *rCYP3A4*	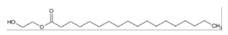	Non-ionic	[61]
Pluronic F68	Midazolam	Strong inhibition of *rCYP3A4*	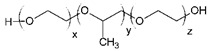	Non-ionic	[61]

**Table 3:** Different types of polymers with some examples.

Polymers	Examples
Natural	Sodium alginate Gelatin Chitosan
Semi-synthetic	Cellulose derivatives
Synthetic	Polyethylene glycols Poloxamers Polyactides Polyamides Acrylic acid polymers
Fermentation products	Xanthan gum

**Table 4:** Effect of different polymers on activities of CYP 2E1, 3A5, 2C9, 2C19, 1A2 and 2D6.

Polymer	IC50 Values (μM)						
	*CYP2E1*	*CYP3A4*	*CYP3A5*	*CYP2C9*	*CYP2C19*	*CYP1A2*	*CYP2D6*
PEG	75.3 ± 2.1	-	78.0 ± 17.8	365.6 ± 32.8	139.0 ± 22.4	-	409.6 ± 34.5
F68	203.7 ± 48.3	59.1 ± 13.6	209.9 ± 29.7	244.8 ± 13.2	-	-	-
F127	218.9 ± 13.3	-	-	-	-	-	-
NaCMC	-	-	-	-	-	224.7 ± 14.8	
HPC	-	-	-	-	-	-	-
HPMC	253.5 ± 17.9	-	19.4 ± 0.6	-	-	-	-
PVA	548.9 ± 30.4	-	-	-	-	-	-
Kollicoat	598.1 ± 26.1	-	-	-	-	10.0 ± 3.9	89.9 ± 2.9
HG	141.2 ± 14.1	-	-	-	-	40.9 ± 8.4	-
PVP	-	107.3 ± 11.2	-	-	-	78.3 ± 4.2	-

**Table 5:** Effect of fatty acids on nine CYPs: 1A2, 2A6, 2B6, 2C8, 2C9, 2C19, 2E1 and 3A4.

Fatty Acid	Absolute IC_50_ (μM)								
	1A2	2A6	2B6	2C8	2C9	2C19	2D6	2E1	3A4
Arachidonic acid	9.7	21.4	4.6	1.3	3.3	23.2	18.3	61.7	11.7
Behenic acid	>30	>30	>30	>30	>30	>30	>30	>30	>30
Cervonic acid	6.3	11.7	6.7	1.2	2.6	15.8	5.6	44.4	7.5
Gondoic acid	16.2	81.9	16.6	6.0	17	>100	>100	>100	>100
Lauric acid	>100	>100	21.5	42.9	>100	>100	>100	>100	>100
Linoleic acid	13.3	28.9	7.1	1.0	7.4	55.8	17.5	58.9	18.5
α-Linolenic acid	8.8	13.5	9.7	4.4	10.6	53.3	34.3	67.2	36.9
Myristic acid	15.8	>100	10.7	13.3	36.1	>100	>100	>100	>100
Nervonic acid	>11.1	>11.1	>11.1	>11.1	>11.1	>11.1	>11.1	>11.1	>11.1
Oleic acid	11.2	25	8.2	4.4	5.7	98.9	18.1	83.8	11.4
Palmitic acid	>100	>100	90.5	>100	>100	>100	>100	>100	>100
Palmitoleic acid	7.8	36.2	8	9.7	11.9	58.1	30.3	72.1	26.5
Stearic acid	>33.3	>33.3	>33.3	>33.3	>33.3	>33.3	>33.3	>33.3	>33.3
Timnodonic acid	8.2	17.4	5.9	1.5	3.8	13.8	5.7	77.4	16

**Table 6:** Effect of saturated and unsaturated fatty acids where, saturated fatty acids showed NI (no inhibition) and unsaturated inhibited all CYP isoforms.

Fatty Acids	IC_50_					
	*CYP1A2*	*CYP2C9*	*CYP2C19*	*CYP2D6*	*CYP2E1*	*CYP3A4*
Palmitic acid	NI	NI	NI	NI	NI	NI
Stearic acid	NI	NI	NI	NI	NI	NI
Linoleic acid	74	4.1	15	192	113	49
Linolenic acid	52	8.1	9.3	151	82	61
Arachidonic acid	37	3.5	4.8	113	67	48
Eicosapentaenoic acid	41	4.4	4.4	127	53	54
Docosahexaenoic acid	41	2.9	6.7	122	65	34

**Table 7:** Effect of excipients on PXR activation, *CYP3A4* and *MDR1*. change.

Excipients	*Fa2N4*		HPH		LS174T	
	mRNA	Protein	mRNA	Protein	*CYP3A4*	*MDR1*
HPMC	↑	↓	=	x	↓	↓a
Pregelatinized starch	=	=	↓	x	↓	↓
Croscarmellose sodium	↑	=	↑	x	↓a	↓a
Crospovidone	↑a	↓	=	x	↓	↓a
Polysorbate-80	↓	↓	↓	↓	=	=

All the excipients failed to activate PXR but reduced expression of *CYP3A4* and *MDR1*. ↑: increase in expression, ↓: decrease in expression, =: no change, x: not measured, a: no significant.

## Abbreviations

API	Active Pharmaceutical Ingredient
AUC	Area Under the Curve
CMC	Critical Micellar Concentration
CTAB	Cetyltrimethylammonium Bromide
CYP450	Cytochrome P450
DHB	Dihydroxybergamottin
DMSO	Dimethyl Sulfoxide
FA	Fatty Acids
GIT	Gastrointestinal Tract
HPC	Hydroxypropyl Cellulose
HPMC	Hydroxypropyl Methylcellulose
IC50	Inhibitory Concentration
Ki	Inhibitory Constant
Clint	Intrinsic Clearance
mRNA	Messenger RNA
MDZ	Midazolam
Mw	Molecular weight
MDR1	Multi-drug Resistance Gene
mPEGx-PCLx	Methoxy Poly(ethylene glycol)-poly(ε-caprolactone) (mPEGx-PCLx)
ATBC	Acetyl Tributyl Citrate
F68	Pluronic F68
PEG	Polyethylene Glycol
PVA	Polyvinyl Acetate
PVP	Polyvinyl Pyrrolidone
PXR	Pregnane X receptor
SDS	Sodium Dodecyl Sulfate
SEDSS	Self-emulsifying Drug Delivery System
SMEDDS	Self-micro Emulsifying Drug Delivery System
NaCMC	Sodium Carboxymethyl Cellulose
NI	No Inhibition
TPGS	D -α-Tocopherol Polyethylene Glycol 1000 Succinate
TX-100	Triton X-100
